# Supplementary material for: Infant movement classification through pressure distribution analysis
Source: Commun Med (Lond). 2023 Aug 16;3:112. doi: 10.1038/s43856-023-00342-5 (PMC10432534; doi:10.1038/s43856-023-00342-5)
Supplement: Supplementary file 1 — Supplementary Information [file 43856_2023_342_MOESM1_ESM.pdf]

Infant movement classification through pressure distribution analysis

Tomas Kulvicius,[1,2*~], Dajie Zhang[1,3,4*], Karin Nielsen-Saines[5], Sven Bölte[6,7,8], Marc Kraft[9], Christa Einspieler[4], Luise Poustka[1,3], Florentin Wörgötter[3,9+], Peter B Marschik[1,3,4,6+]

[1]Child and Adolescent Psychiatry and Psychotherapy, University Medical Center Göttingen, Göttingen, Germany
[2]Department for Computational Neuroscience, Third Institute of Physics-Biophysics, Georg-August-University of Göttingen, Göttingen, Germany
[3]Leibniz-ScienceCampus Primate Cognition, Göttingen, Germany
[4]iDN – interdisciplinary Developmental Neuroscience, Division of Phoniatrics, Medical University of Graz, Graz, Austria
[5]Division of Pediatric Infectious Diseases, David Geffen UCLA School of Medicine, Los Angeles, USA
[6]Center of Neurodevelopmental Disorders (KIND), Centre for Psychiatry Research, Department of Women's and Children's Health, Karolinska Institutet, Stockholm, Sweden
[7]Child and Adolescent Psychiatry, Stockholm Health Care Services, Region Stockholm, Stockholm, Sweden
[8]Curtin Autism Research Group, Curtin School of Allied Health, Curtin University, Perth, Western Australia
[9]Department of Medical Engineering, Technical University Berlin, Berlin, Germany
~Corresponding author (E-mail: tomas.kulvicius@uni-goettingen.de)
*These authors contributed equally
[+]These authors jointly supervised this work

Supplemental material

Supplementary Table 1: Details of the Support Vector Machine (SVM), Feed Forward Network (FFN), Convolutional Neural Network (CNN), and Long Short-Term Memory (LSTM) network architectures. After each fully connected (FC), convolutional layer (Conv) and LSTM layer, a batch normalisation layer, and a drop-out layer (10%) were used. ReLU activation functions were used in the FC, Conv and LSTM layers, whereas in the output layer a sigmoid activation function was used.

SVM								
Name	Input dim.	Kernel		Output dimension				
S1.RBF	12	Radial basis function		1				
S1.P1	12	Linear polynomial		1				
S1.P2	12	Quadratic polynomial		1				
S1.P3	12	Cubic polynomial		1				
S2.RBF	24	Radial basis function		1				
S2.P1	24	Linear polynomial		1				
S2.P2	24	Quadratic polynomial		1				
S2.P3	24	Cubic polynomial		1				
FFN								
Name	Input dim.	FC 1 neurons	FC 2 neurons	Output dimension				
F1.1	12	100	-	1				
F1.2	24	100	-	1				
F1.3	24	200	-	1				
F2	24	200	100	1				
CNN								
Name	Input dim.	Conv 1 filters / size	Conv 2 filters / size	Conv 3 filters / size	Average pooling	FC 1 neurons	FC 2 neurons	Output dim.
C1F1.1	500x6	4 / 7x1	-	-	500x1	100	-	1
C1F1.2	500x6	16 / 13x1	-	-	500x1	100	-	1
C1F1.3	500x6	64 / 21x1	-	-	500x1	100	-	1
C1F1.4	500x6	64 / 21x1	-	-	500x1	200	-	1
C1F2	500x6	64 / 21x1	-	-	500x1	200	100	1
C2F1	500x6	4 / 7x1	16 / 13x1	-	500x1	100	-	1
C3F1.1	500x6	4 / 7x1	16 / 13x1	64 / 21x1	500x1	100	-	1
C3F1.2	500x6	4 / 7x1	16 / 13x1	64 / 21x1	500x1	200	-	1
C3F2	500x6	4 / 7x1	16 / 13x1	64 / 21x1	500x1	200	100	1
LSTM								
Name	Input dim.	LSTM 1 neurons	LSTM 2 neurons	FC 1 neurons	FC 2 neurons	Output dim.		
L1F1.1	25x120	64	-	100	-	1		
L1F1.2	50x60	64	-	100	-	1		
L1F1.3	100x30	64	-	100	-	1		
L1F1.4	50x60	64	-	200	-	1		
L1F2	50x60	64	-	200	100	1		
L2F2.1	50x60	64	32	200	100	1		
L2F2.2	50x60	128	64	200	100	1		

Supplementary Table 2: Comparison of different classifiers (see Supplementary Table 1) on classification of the presence vs. absence of fidgety movements.

Average sensitivity, specificity and balanced accuracy is shown obtained on five test sets. Numbers in brackets correspond to confidence intervals of the mean (CI 95%). Numbers in bold font correspond to the accuracies of the best models. Raw data is available in Supplementary Data.

Classifier	Sensitivity (%)	Specificity (%)	Balanced Accuracy (%)
SVM			
S1.RBF	73.15 [68.09 78.20]	69.83 [62.39 77.27]	71.49 [69.70 73.28]
S1.P1	72.10 [66.46 77.73]	69.95 [60.13 79.77]	71.03 [68.77 73.28]
S1.P2	72.30 [66.64 77.96]	68.74 [58.94 78.53]	70.52 [68.24 72.80]
S1.P3	72.92 [68.40 77.43]	65.35 [57.51 73.19]	69.13 [66.63 71.63]
S2.RBF	74.72 [70.68 78.77]	73.01 [66.51 79.52]	73.87 [70.92 76.82]
S2.P1	78.60 [76.80 80.41]	73.70 [65.65 81.75]	**76.15** [72.00 80.30]
S2.P2	80.49 [77.56 83.43]	71.54 [65.27 77.82]	76.02 [72.71 79.32]
S2.P3	79.04 [75.77 82.30]	73.12 [67.39 78.85]	76.08 [74.57 77.58]
FFN			
F1.1	73.95 [70.60 77.29]	70.27 [59.16 81.39]	72.11 [68.11 76.11]
F1.2	81.32 [78.46 84.17]	68.29 [60.89 75.69]	74.80 [71.16 78.45]
F1.3	79.96 [77.11 82.82]	71.18 [63.44 78.92]	**75.57** [72.65 78.49]
F2	79.90 [74.02 85.78]	67.25 [54.69 79.81]	73.58 [69.52 77.63]
CNN			
C1F1.1	85.35 [82.47 88.22]	69.58 [62.03 77.13]	77.46 [74.09 80.84]
C1F1.2	81.66 [76.69 86.63]	68.04 [58.18 77.90]	74.85 [71.15 78.55]
C1F1.3	80.57 [78.10 83.04]	69.48 [64.00 74.96]	75.03 [71.54 78.51]
C1F1.4	78.03 [74.10 81.96]	69.82 [62.59 77.05]	73.93 [70.36 77.49]
C1F2	79.26 [75.62 82.90]	72.75 [65.08 80.41]	76.00 [72.93 79.08]
C2F1	84.42 [80.15 88.69]	74.30 [62.43 86.16]	79.36 [73.84 84.88]
C3F1.1	84.48 [79.25 89.70]	75.71 [68.62 82.80]	80.09 [77.37 82.81]
C3F1.2	86.06 [81.89 90.24]	74.80 [67.72 81.89]	80.43 [76.70 84.17]
C3F2	86.48 [82.92 90.03]	76.37 [70.71 82.04]	**81.43** [78.00 84.86]
LSTM			
L1F1.1	69.54 [66.14 72.94]	64.59 [56.34 72.83]	67.06 [63.85 70.28]
L1F1.2	77.89 [73.67 82.12]	60.14 [50.85 69.42]	69.01 [65.44 72.59]
L1F1.3	76.10 [71.00 81.20]	57.75 [50.24 65.25]	66.93 [64.91 68.95]
L1F1.4	75.11 [69.23 80.98]	61.97 [51.86 72.08]	68.54 [63.74 73.33]
L1F2	78.13 [73.97 82.30]	59.94 [53.60 66.29]	**69.04** [65.94 72.13]
L2F2.1	82.21 [76.24 88.19]	51.99 [34.45 69.54]	67.10 [61.06 73.15]
L2F2.2	75.11 [69.23 80.98]	61.97 [51.86 72.08]	68.54 [63.74 73.33]
