# Supplementary material for: Infant movement classification through pressure distribution analysis
Source: Commun Med (Lond). 2023 Aug 16;3:112. doi: 10.1038/s43856-023-00342-5 (PMC10432534; doi:10.1038/s43856-023-00342-5)
Supplement: Supplementary file 3 — Reporting Summary [file 43856_2023_342_MOESM3_ESM.pdf]

nature portfolio

Corresponding author(s): Tomas Kulvicius

Last updated by author(s): Jul 7, 2023

Reporting Summary

Nature Portfolio wishes to improve the reproducibility of the work that we publish. This form provides structure for consistency and transparency in reporting. For further information on Nature Portfolio policies, see our Editorial Policies and the Editorial Policy Checklist.

Statistics

For all statistical analyses, confirm that the following items are present in the figure legend, table legend, main text, or Methods section.

n/a	Confirmed	
☐	☒	The exact sample size (*n*) for each experimental group/condition, given as a discrete number and unit of measurement
☒	☐	A statement on whether measurements were taken from distinct samples or whether the same sample was measured repeatedly
☐	☒	The statistical test(s) used AND whether they are one- or two-sided<br>*Only common tests should be described solely by name; describe more complex techniques in the Methods section.*
☒	☐	A description of all covariates tested
☒	☐	A description of any assumptions or corrections, such as tests of normality and adjustment for multiple comparisons
☐	☒	A full description of the statistical parameters including central tendency (e.g. means) or other basic estimates (e.g. regression coefficient) AND variation (e.g. standard deviation) or associated estimates of uncertainty (e.g. confidence intervals)
☐	☒	For null hypothesis testing, the test statistic (e.g. *F*, *t*, *r*) with confidence intervals, effect sizes, degrees of freedom and *P* value noted<br>*Give P values as exact values whenever suitable.*
☒	☐	For Bayesian analysis, information on the choice of priors and Markov chain Monte Carlo settings
☒	☐	For hierarchical and complex designs, identification of the appropriate level for tests and full reporting of outcomes
☒	☐	Estimates of effect sizes (e.g. Cohen's *d*, Pearson's *r*), indicating how they were calculated

Our web collection on statistics for biologists contains articles on many of the points above.

Software and code

Policy information about availability of computer code

Data collection	Custom code was used to perform recordings and obtain data.
Data analysis	Matlab R2019b, Python 3.7.6

For manuscripts utilizing custom algorithms or software that are central to the research but not yet described in published literature, software must be made available to editors and reviewers. We strongly encourage code deposition in a community repository (e.g. GitHub). See the Nature Portfolio guidelines for submitting code & software for further information.

Data

Policy information about availability of data

All manuscripts must include a data availability statement. This statement should provide the following information, where applicable:

- Accession codes, unique identifiers, or web links for publicly available datasets
- A description of any restrictions on data availability
- For clinical datasets or third party data, please ensure that the statement adheres to our policy

Data and code is publicly available at Zenodo: https://doi.org/10.5281/zenodo.8104097.

Human research participants

Policy information about studies involving human research participants and Sex and Gender in Research.

Reporting on sex and gender	23 females and 22 males
Population characteristics	Data obtained from 45 neurotypical infants were used. Data stem from prospective longitudinal assessments between 4 and 16 weeks post-term age. Included infants had available video recordings, accelerometer data, audio- and pressure sensor data of spontaneous neuromotor functions recorded in biweekly intervals. In this study only pressure sensor data was used.
Recruitment	Paticipants were recruited through website advertisements, mail blasts, facebook and social media postings, existing databases at the University. No sampling bias expected.
Ethics oversight	Approved by the Institutional Review Board of the Medical University of Graz, Austria (27-476ex14/15) and the University Medical Center Göttingen, Germany (20/9/19).

Note that full information on the approval of the study protocol must also be provided in the manuscript.

Field-specific reporting

Please select the one below that is the best fit for your research. If you are not sure, read the appropriate sections before making your selection.

☐ Life sciences ☒ Behavioural & social sciences ☐ Ecological, evolutionary & environmental sciences

For a reference copy of the document with all sections, see nature.com/documents/nr-reporting-summary-flat.pdf

Behavioural & social sciences study design

All studies must disclose on these points even when the disclosure is negative.

Study description	We used a pressure sensing mat for movement classification to distinguish presence vs. absence of fidgety movements according to general movement assessment (GMA).
Research sample	Data from a prospective longitudinal cohort of 45 (23 females and 22 males) typically developing infants between 4 and 16 weeks post-term age were analysed. Data acquisition was conducted at iDN's BRAINtegrity lab at the Medical University of Graz, Austria.
Sampling strategy	We segmented the data stream into 5-second snippets. From a total of 19451 available snippets, 2800 were randomly selected for GMA which was was performed by to senior assessors. For movement classification using machine learning 1776 snippets with matched ratings from both assessors were used (828 with absence of fidgety movements and 948 with presence of fidgety movements).
Data collection	Pressure data was acquired using a Conformat pressure sensing mat (Tekscan, Inc., South Boston, Massachusetts, USA5. This mat contains 1024 pressure sensors arranged in a 32 x 32 array on an area of 471.4 x 471.4 mm2, producing pressure image frames (8 Bit, 32 x 32 pixels, sampling rate 100 Hz).
Timing	Data collection, segmentation, annotation and analyses took place from 2017 to 2021.
Data exclusions	See sampling strategy above.
Non-participation	n/a
Randomization	See sampling strategy above.

Reporting for specific materials, systems and methods

We require information from authors about some types of materials, experimental systems and methods used in many studies. Here, indicate whether each material, system or method listed is relevant to your study. If you are not sure if a list item applies to your research, read the appropriate section before selecting a response.

Materials & experimental systems

n/a	Involved in the study
☒	☐ Antibodies
☒	☐ Eukaryotic cell lines
☒	☐ Palaeontology and archaeology
☒	☐ Animals and other organisms
☒	☐ Clinical data
☒	☐ Dual use research of concern

Methods

n/a	Involved in the study
☒	☐ ChIP-seq
☒	☐ Flow cytometry
☒	☐ MRI-based neuroimaging
