# Supplementary material for: Infant movement classification through pressure distribution analysis
Source: Commun Med (Lond). 2023 Aug 16;3:112. doi: 10.1038/s43856-023-00342-5 (PMC10432534; doi:10.1038/s43856-023-00342-5)
Supplement: Supplementary file 4 — Description of Additional Supplementary Files [file 43856_2023_342_MOESM4_ESM.pdf]

Description of Additional Supplementary Files

File Name: Supplementary Data

Description: The source data underlying results presented in Figure 5 and Supplementary Table 2
